# Monitoring Work Ability Index During a Two-Year Period Among Portuguese Municipality Workers

**DOI:** 10.3390/ijerph16193674

**Published:** 2019-09-30

**Authors:** Teresa Patrone Cotrim, Camila Ribeiro, Júlia Teles, Vítor Reis, Maria João Guerreiro, Ana Sofia Janicas, Susana Candeias, Margarida Costa

**Affiliations:** 1 Ergonomics Laboratory, Faculdade de Motricidade Humana, Universidade de Lisboa, 1499-002 Lisbon, Portugal; camiladriribeiro@gmail.com (C.R.); jteles@fmh.ulisboa.pt (J.T.); 2 CIAUD, Faculdade de Arquitetura, Universidade de Lisboa, 1349-063 Lisbon, Portugal; 3 CIPER, Faculdade de Motricidade Humana, Universidade de Lisboa, 1499-002 Lisbon, Portugal; 4 Health and Safety Department, Municipality of Sintra, 2710-437 Sintra, Portugal; vreis@cm-sintra.pt (V.R.); maria.guerreiro@cm-sintra.pt (M.J.G.); ana.janicas@cm-sintra.pt (A.S.J.); susana.candeias@cm-sintra.pt (S.C.); maria.costa@cm-sintra.pt (M.C.)

**Keywords:** WAI, municipal workers, prospective study, COPSOQ II, predictive factors

## Abstract

In Portugal, little is known about the work ability profiles of municipal workers and their changes during working life. In order to characterize and understand the changes in work ability among municipal workers, a prospective study was designed to begin in 2015 in the municipality of Sintra, in the surroundings of Lisbon, and to collect data every two years. The present paper aims at characterizing the changes in the work ability of those workers between 2015 and 2017 and to identify the main predictors. Data collection was based on a questionnaire that encompassed socio-demographic data, the Copenhagen Psychosocial Questionnaire II (COPSOQ II), the Nordic questionnaire adapted, and the Work Ability Index (WAI). In this two-year period, the work ability of municipal workers decreased and the main predictive factors were age, lower-back pain, negative health perception, the presence of burnout, and making manual efforts. Still, there were factors that act as positive predictors of an excellent work ability, such as having training in the previous two years, a good sense of community at work, and a favorable meaning of work. In summary, the intervention strategies in the work field should take into consideration the main predictors of work ability that are relevant for each organization.

## 1. Introduction

Between 2009 and 2016, the economic crisis in Portugal raised severe restrictions to public administration. Several measures applied to the public sector, such as salary cuts, reduction in overtime compensation, suspension of several public holidays, reduction in the number of vacation days, and an increase in weekly working hours from 35 h to 40 h [1], affected municipal workers and changed the well-being in municipalities. In addition, it is described in European Countries that the crisis increased job insecurity and job dissatisfaction, impacting work-related stress and mental health. Also, the self-perceived poor health status increased during the crisis period [2].

Furthermore, in 2014 the retirement age in the public sector increased from 65 to 66, due to changes in the sustainability factor. This new mechanism has been enshrined into legislation to increase the retirement age. In the meantime, the reference salary for pension calculation was adjusted [1]. These measures promoted the permanence of municipal workers in their jobs until older ages.

All of these changes have impacted the working and personal life of municipal workers, contributing to changes in their work ability perception. Work ability is based on the balance between the individuals’ resources and work demands [3] and is strongly determined by individual factors, such as health status, lifestyle, work demands, and physical, organizational, or psychological conditions [4,5].

Municipal workers have been studied in Finland, since the decade of 1980, allowing to follow the work ability trajectories and the main determinants that influence its changes [4,6,7,8].

In Portugal, little is known about the work ability profiles of municipal workers and their changes during working life [9,10]. Even though the present study started in 2015, it was considered relevant to analyze the impact of individual and work determinants on work ability among Portuguese municipal workers during the final period of the financial crisis in Portugal, and to monitor the changes every two years. Starting in 2015, it could be expected that the individual manifestations of the impact of the crisis related to the period between 2009 and 2015 showed up like described by Mucci et al. (2016) [2]: Job insecurity, job dissatisfaction, work-related stress, and poor self-perceived health, among others. There is evidence that psychosocial factors influence work ability [4,5] but also the perception of physical well-being [11] that, in turn, influences work ability. Some studies showed that the effect of stress and pain on work ability were additive [11], which stresses the need to evaluate both when determining the main predictors of work ability.

The work ability concept considers the balance between individual and work factors [3] and it is operationalized by the Work Ability Index questionnaire [8,12,13], but psychosocial factors can be addressed by the Copenhagen Psychosocial Questionnaire.

In order to characterize and understand the changes in work ability among municipal workers, a prospective study was designed to begin in 2015 in the municipality of Sintra, in the surroundings of Lisbon, and to collect data every two years. The present paper aims at characterizing the changes in the work ability of those workers between 2015 and 2017 and to identify the main predictors.

## 2. Materials and Methods

The study design was prospective, based on a survey applied to municipal workers, between May and June of 2015 and in the same period of 2017. The survey followed a paper and pencil format. The workers were contacted personally, and informed about the study design and its objectives. The questionnaire was applied further to a written informed consent explaining that participation in the study was voluntary and anonymous. In October of 2014, the study was approved by the mayor of the Municipality of Sintra, and in May of 2015, by the ethical committee of the Human Kinetics Faculty.

### 2.1. Participants

The population of municipal workers was stable during the time frame of the study, and consisted of 1667 workers during both years. The inclusion criteria were to have been working in the municipality for at least six months, to voluntarily answer the questionnaire, and to have answered all items of the Work Ability Index allowing for the calculation of the final score. The response rate was 52.1% (*n* = 868) in 2015, and 68.4% (*n* = 1140) in 2017. Due to the lack of permission, it was not possible to code the questionnaires in the two evaluated years, so the two samples were independent and the questionnaires completely anonymous. The increase in the response rates was understandable because the two samples were not paired, and there was an increased awareness of the study objectives based on the information activities developed during the two-year period.

### 2.2. Variables

The outcome variable was the work ability perception measured by the Work Ability Index (WAI) [8,12]. The explanatory variables were selected based on previously described associations with work ability [4,11,14,15], such as the psychosocial factors measured by the Copenhagen Psychosocial Questionnaire II (COPSOQ II); the individual factors such as age, work seniority, gender, qualifications, and the mean duration of sleep hours; the physical determinants of work, such as training and work accidents in the previous two years, fatigue perception, repetitiveness of hand movements, manual efforts, and manual materials handling; and musculoskeletal symptomatology.

### 2.3. Research Questions

The main research question is: What are the main predictors of an excellent work ability among municipal workers?

Additionally, there were questions raised about the differences between 2015 and 2017 concerning: Socio-demographic variables, physical work characteristics, musculoskeletal symptoms, and psychosocial factors.

### 2.4. Questionnaire

The questionnaire was developed according to the study aims and the literature review. It included three parts: Questions regarding socio-demographic characterization and determinants of work, the Portuguese medium version of the Copenhagen Psychosocial Questionnaire II (COPSOQ II) [16], and the Portuguese version of the Work Ability Index [17].

The first part of the questionnaire included socio-demographic data such as age, work seniority, gender, qualifications, and the mean duration of sleep hours; the data regarding the physical determinants of work, such as training and work accidents in the previous two years, fatigue perception, repetitiveness of hand movements, manual efforts, and manual materials handling; and an adaptation of the Nordic questionnaire in order to characterize musculoskeletal symptomatology in the last twelve months [18].

The Portuguese medium version of the Copenhagen Psychosocial Questionnaire II (COPSOQ II) [16] was used to assess the psychosocial risk factors. The COPSOQ II is a standardized questionnaire covering a broad range of psychosocial factors [19,20]. The results of each scale were analyzed using a range of points from 1 to 5, where 1 represents minimum risk, and 5 maximum risk.

The Portuguese version of the Work Ability Index was used to describe the workers’ assessment regarding their own work ability [17]. WAI includes seven items, namely actual work ability, physical and mental work demands, diagnosed illnesses, work limitations due to illness, absenteeism, work ability prognosis, and psychological resources. The WAI final score allows to classify work ability into poor (7–27), moderate (28–36), good (37–43), or excellent (44–49) [17].

### 2.5. Statistical Procedures

The 5-point (1–5) Likert scales were grouped in two or three categories in order to allow the implementation of the logistic regression model: The repetitiveness of hand movements, manual efforts, and manual materials handling variables were grouped in three categories (never/seldom, sometimes, frequent/very frequent); shoulder, elbow, and wrist symptomatology was dichotomized into presence (yes) or absence (no) of the symptoms. Age was dichotomized into below and above 50 years old.

The scores of the COPSOQ II scales were described using the mean and standard deviation.

The WAI was analyzed using the four categories: poor and moderate (unsatisfactory level), and good and excellent, corresponding to the satisfactory level of work ability. The level of good work ability is commonly the most prevalent [7,10,21,22,23,24], so this category was excluded from the logistic regression analysis.

The differences in the variables of the study, between 2015 and 2017, were analyzed using independent samples *t*-tests and chi-square tests of homogeneity for quantitative and qualitative variables, respectively.

A logistic regression model considering WAI (1 = excellent, 0 = unsatisfactory) as the dependent variable was adjusted, meaning that the model estimates the probability of a municipal worker having an excellent WAI. The backward stepwise method using the Wald statistic was applied for the model variable selection procedure. The independent variables selected for the model were: Date, age, lower-back symptoms, burnout, global health perception, training in the last two years, manual efforts, sense of community at work, and meaning of work. For the continuous predictors, the linearity in the logit was verified. To assess the fit of the models, several goodness-of-fit measures were calculated. In particular, the area under the receiver operating characteristic (ROC) curve (AUC) to evaluate the model’s predictive accuracy.

## 3. Results

This section includes the presentation and analysis of the results of the socio-demographic and work-related factors, the COPSOQ II scales, the WAI categories, and the predictors of the WAI.

### 3.1. Sociodemographics and Work-Related Characteristics

The participants had a mean age of 46.9 years (SD = 8.2) in 2015 and 48.4 years (SD = 8.7) in 2017, and the difference was statistically significant (*p* ≤ 0.001). Work seniority was higher in 2015 (20.3 ± 8.6) then in 2017 (19.3 ± 9.8), and the difference was also statistically significant (*p* = 0.023) (Table 1). The two variables were correlated in both years (2015: r = 0.615 *p* = 0.010; 2017: r = 0.617 *p* = 0.011). In the logistic regression analysis, age was selected to be included in the model, in detriment of work seniority.

In both years, the participants were mainly women, under the age of 50 years old, having completed high school (Table 2).

Regarding the work-related factors, in both years, the majority of the participants had training and had no work accidents in the previous two years, had frequent or very frequent repetitiveness of hand movements, and seldomly or never made manual efforts and manual materials handling (Table 3). When comparing the variables’ repetitiveness of hand movements, manual efforts and manual materials handling between 2015 and 2017, it was found that the category “never/seldom” obtained higher percentages in the year 2017, and that the difference was statistically significant (*p* < 0.050). This can be explained by an increase in the participation of white-collar workers in the 2017’ sample (Table 3).

The prevalence of self-reported symptoms was higher in 2017 for all the regions, with an exception made for the wrists, but only for the shoulder region was the difference statistically significant (*p* = 0.009). The self-reported musculoskeletal symptoms in the last 12 months were reported with a higher frequency for the lower-back region for both years (Table 4).

### 3.2. Psychosocial Factors—COPSOQ II

Regarding the scales of the COPSOQ II for which the higher values are unfavorable, the worse results in 2015 were found for pace of work, cognitive demands, emotional demands, and job insecurity. The results of these scales got better from 2015 to 2017, and the differences were statistically significant, with an exception made for the cognitive demands scale which maintained the same level of risk. The scales of role conflicts and horizontal trust had intermediate levels in both years, but in 2017 the results were better and the differences were statistically significant. The health-related scales had intermediate levels in both years, but the levels of stress, burnout, and depressive symptoms got lower in 2017 (Table 5).

With respect to the scales of the COPSOQ II for which the higher values are favorable, the best results in 2015 were found for role clarity and sense of community at work, and these results were maintained at similar levels in 2017. The results of the scales that got better from 2015 to 2017 with statistically significant differences were predictability, recognition/rewards, support from superiors, quality of leadership, vertical trust, organizational justice, meaning of work, and work satisfaction (Table 6).

### 3.3. Work Ability Index

The mean results of the WAI decreased slightly from 2015 (40.7 ± 5.1; 14−49) to 2017 (40.2 ± 5.1; 7−49), but the difference was statistically significant (*p* = 0.016). When looking at the distribution by categories, it is possible to understand these changes. The results show that most of the participants had good work ability in both years, with a similar percentage. The two categories that have changed from 2015 to 2017 were the moderate and the excellent ones. The moderate work ability category increased from 2015 to 2017 and the excellent work ability category decreased during the same period (Table 7). The difference in the work ability distribution between the two years was statistically significant (χ^2^ (3) = 7.483; *p* = 0.006).

### 3.4. Predictors of WAI

According to the model, the log of the odds of a municipal worker who had an excellent WAI was: Negatively related with date, age, having lower-back symptoms in the last 12 months, burnout, and having unfavorable global health perception; and positively related with having favorable global health perception, having training in the last 2 years, rarely or never making manual efforts, sense of community at work, and meaning of work (Table 8).

The odds of a municipal worker who had an excellent WAI (compared with an unsatisfactory WAI) decreased: 2.0 times for workers aged ≥50 years; 2.7 times for the two-year period (i.e., from 2015 to 2017); 3.2 times for those who reported lower-back symptoms in the last 12 months; 2.7 times for each unit of increase in burnout; and 13.7 times for workers with unfavorable global health (compared to those who had an intermediate global health) (Table 8).

The odds of a municipal worker who had an excellent WAI (compared with an unsatisfactory WAI) increased: 7.5 times for workers with favorable global health (compared to those who had intermediate global health); 1.8 times for those who had training in the last two years; 3.1 times for those who reported never or rarely making manual efforts; 1.9 times for each unit of increase in sense of community at work; 1.9 times for each unit of increase in meaning of work (Table 8).

The logistic regression results are shown in Table 8. The area under the ROC curve (AUC = 0.950) showed that the model has good predictive accuracy, i.e., has the ability to distinguish municipal workers with an excellent WAI from those who have unsatisfactory WAI.

## 4. Discussion

As far as it is known, this is the first prospective study done in Portugal focused on the characterization of the Work Ability Index and its determinants among municipal workers [9,10]. The collection of data regarding the work ability index over the years provides first-hand knowledge of the determinants of the WAI and its changes, which can be considered one of the major contributions of the study. In the literature, most of the longitudinal studies regarding work-related characteristics and the WAI were done with municipal workers from Finland [4,7,8,25], but other occupations have also been studied [5,14,15,26,27,28]. The main determinants of the WAI that showed up from the literature were age, lower education level, poor musculoskeletal capacity, poor health, psychosocial factors (poor management, poor satisfaction with the supervisor), and high physical demands (increased muscular work, poor work postures) [3,4,5,6,7,12,15,25,26,27].

Globally, in Portugal, the population is aging, and the working population is also aging and decreasing. Between 2012 and 2017, the working age population (15 to 64 years of age) was reduced from 65.8% to 64.7%, and the percentage of elderly population (65 years of age and older) increased from 19.4% to 21.5%. The ageing index changed from 131.1 to 155.4 elderly people per 100 young people [29]. These changes create huge pressure on the working population. Changes in Portuguese regulations in 2014 led to a raise in the retirement age in the public sector from 65 to 66 years old [1]. Municipal workers also suffered with the financial crisis that Portugal faced from 2008 until recent years because several measures were applied to the public sector [1], affecting the well-being in municipalities.

The results of our study are in line with these changes. In 2015, the mean age of our sample of municipal workers was 47 years old, and in 2017 it raised to 48.4 years, with an increase in the percentage of workers above 50 years old. Age appeared as one of the main predictors of the WAI. According to the model, the log of the odds of a municipal worker who had an excellent WAI was negatively related with age. The odds of a municipal worker who had an excellent WAI decreased 2.0 times for workers older than 50 years, which is a common finding in other studies [5,30] with the WAI having a strong decline over 50 years [26,31]. Nevertheless, different paths across the working life may influence the changes in work ability in the long run due to the presence of work strain in different moments of working lives [4].

The absence of high physical demands is a predictor of better work ability, with these workers having 3.1 more chances of having an excellent WAI when compared with those making manual efforts frequently or very frequently. High physical work demands are a well-known factor that contributes to a lower work ability [3,4,7,25], and for workers over 50 years old determining recurrently the drop out of an active working live [11]. At the same time, repetitive movements, awkward postures, and forceful exertion are associated with an increased risk of musculoskeletal disorders among the middle-aged groups [32,33].

Health factors, such as work-related musculoskeletal disorders and mental strain, are strong predictors of lower work ability [4,25,34,35]. Among our sample of municipal workers, lower-back symptoms varied from 45% in 2015 to 49% in 2017, although the difference was not statistically significant, and appeared as an important predictor of lower work ability. For those reporting lower-back symptoms, the chance of having an excellent WAI decreased 3.2 times. Additionally, the perception of burnout and a negative global health perception were also negatively related to the chance of having an excellent WAI. The chance of having an excellent WAI decreased 13.7 times for those reporting a negative health perception, and 2.7 times for each unit of increase in burnout perception. Some studies showed that stress and pain had an additive effect on work ability [11], determining its decrease.

Psychosocial factors, such as low job control, low social support, low reward relative to effort, and work–family conflict, are also addressed in several studies as being related with poor WAI [5,14,25]. In our sample of municipal workers, an improvement in the majority of the COPSOQ II scales was found from 2015 to 2017. This can be understood based on the formal end of the financial crisis in Portugal and the withdrawal of the strict measures that affected municipal workers and their families. Sense of community at work was one of the scales with the best results during the two years; this scale, together with meaning of work, appeared as positive predictors of an excellent work ability. These scales may act as protective factors regarding work ability along the years.

Additionally, having had training in the last two years increased the chance of an excellent work ability by 1.8 times. In some studies, training had a positive influence on psychological well-being and on the acquisition of competencies [36,37], which supported a better WAI [38]. Training makes the workers more motivated and flexible, and predisposes them towards greater mobility in the organizations promoting their employability [36].

From an ergonomics point of view, the work demands must be adjusted to the worker capabilities. Work ability is the result of the balance between work demands and individual characteristics [7,12]. In our model, the predictors can be grouped into those related to individual characteristics (age, lower-back symptoms, health and burnout perception, physical demands of work (perception of manual efforts), and organizational characteristics of work (training in the last two years, meaning of work, sense of community at work)).

Our study may have had some limitations because all of the information was collected using a questionnaire, which may lead to a recall bias. All work-related determinants were also measured by the questionnaire, which may have influenced the results when participants with poor WAI overestimated their workload in the workplace [5]. Also, the selection process might have affected our results; because the codification process was not allowed, it was impossible to have paired samples for the two measures. This fact determined independent samples for the two-year follow-up, with a slightly different composition. Participation in the study was more likely to have occurred among workers with more health problems, as well as with higher perceptions of exposure levels [15], which may have led to leaving the healthiest workers out of the study. However, the strengths of the study are related with the prospective design and the large sample size.

This project is still under development and data collection will continue every two years. The findings of the project will support the municipality of Sintra in establishing age-related organizational interventions focused on the identified determinants associated with lower work ability [5]. These measures must be focused on promoting awareness of managers to the importance of the physical and mental health of municipal workers [11,39], decreasing physical and psychosocial demands [5,11], and contributing to the promotion of work ability along the life course [36].

The results of the study may help the managers of the municipality to decide what programs of occupational risks prevention or health promotion must be funded, making an informed decision based on the main predictors of an excellent WAI.

## 5. Conclusions

This study showed that in a two-year period, the work ability of this sample of municipal workers decreased and that the main factors were age, lower-back pain, negative health perception, the presence of burnout, and making manual efforts. Among these factors, some are preventable and must be managed regarding a healthy aging process in work sites. Still, there are factors that should be increased in the future because they act as positive predictors of an excellent work ability, such as having training in the previous two years, a good sense of community at work, and a favorable meaning of work. In summary, the intervention strategies in work fields should be tailored, taking into consideration the main predictors of work ability that are relevant for each organization.

## Figures and Tables

**Table 1:** Age, seniority, sleep hours, and perception of fatigue among study participants in 2015 and 2017.

Socio-Demographic Factors	2015				2017			
	*n*	Min–Max	Mean	SD	*n*	Min–Max	Mean	SD
Age (years)	851	25–69	46.9	8.2	1123	21–68	48.4	8.7
Work seniority (years)	815	1–46	20.3	8.6	977	1–45	19.3	9.8
Sleep Hours	849	4–10	6.8	1.0	1116	4–10	6.8	0.9
Perception of fatigue	838	0–10	6.5	1.7	1085	0–10	6.0	2.0

**Table 2:** Age groups, sex, and qualifications among study participants in 2015 and 2017.

Socio-Demographic Factors		2015		2017	
		*n*	%	*n*	%
Age Groups	<50 years	521	61.2	593	52.8
	≥50 years	330	38.8	530	47.2
Gender	Female	548	65.6	689	61.8
	Male	287	34.4	425	38.2
Qualifications	Elementary/Junior high school	242	28.2	314	27.9
	High school	324	37.8	411	36.5
	Graduate/Postgraduate	291	34.0	402	34.9

**Table 3:** Training in the last two years, work accident in the last two years, repetitiveness of hand movements, manual efforts, manual materials handling below 4 kg, manual materials handling 5–9 kg, and manual materials handling 10–20 kg by the study participants in 2015 and 2017.

Physical Work-Related Factors		2015		2017	
		*n*	%	*n*	%
Training in the last two years	Yes	521	61.2	560	50.1
	No	330	38.8	557	49.9
Work Accident in the last Two years	Yes	68	8.0	94	8.2
	No	783	92.0	1050	91.8
Repetitiveness of hand movements	Never/Seldom	201	26.4	295	29.0
	Sometimes	163	21.4	178	17.5
	Frequent/Very Frequent	397	52.2	545	53.5
Manual Efforts	Never/Seldom	407	53.9	659	66.1
	Sometimes	193	25.6	166	16.6
	Frequent/Very Frequent	155	20.5	172	17.3
Manual materials handling <4 kg	Never/Seldom	305	40.0	532	52.9
	Sometimes	243	31.8	222	22.1
	Frequent/Very Frequent	215	28.2	252	25.0
Manual materials handling 5–9 kg	Never/Seldom	462	60.9	680	68.0
	Sometimes	171	22.5	159	15.9
	Frequent/Very Frequent	126	16.6	161	16.1
Manual materials handling 10–20 kg	Never/Seldom	584	76.9	805	80.6
	Sometimes	98	12.9	103	10.3
	Frequent/Very Frequent	77	10.2	91	9.1

**Table 4:** Musculoskeletal symptoms in the last 12 months among study participants in 2015 and 2017.

Musculoskeletal Symptoms		2015		2017	
		*n*	%	*n*	%
Cervical region	Yes	312	37.4	465	40.6
	No	523	62.6	680	59.4
Dorsal region	Yes	274	32.8	417	36.4
	No	561	67.2	728	63.6
Lower-Back	Yes	374	44.8	563	49.2
	No	461	55.2	582	50.8
Shoulders	Yes	268	32.1	433	37.8
	No	567	67.9	712	62.2
Elbows	Yes	92	11.0	157	13.7
	No	743	89.0	988	86.3
Wrists	Yes	175	21.0	157	13.7
	No	660	79.0	988	86.3

**Table 5:** Psychosocial factors among study participants in 2015 and 2017—COPSOQ II scales for which the higher value are unfavorable.

COPSOQ II	2015				2017			
	*n*	Min–Max	Mean	SD	*n*	Min–Max	Mean	SD
Quantitative Demands	819	1–5	2.30	0.86	1128	1–5	2.28	0.84
Pace of Work *	848	1–5	3.04	1.02	1126	1–5	2.94	1.04
Cognitive Demands	835	1–5	3.54	0.77	1127	1–5	3.55	0.73
Emotional Demands *	852	1–5	3.27	1.18	1126	1–5	3.12	1.15
Role Conflicts *	843	1–5	2.89	0.71	1124	1–5	2.81	0.73
Horizontal Trust *	821	1–5	2.41	0.79	1110	1–5	2.33	0.80
Job Insecurity **	846	1–5	3.34	1.43	1124	1–5	2.84	1.49
Work–Family Conflict	845	1–5	2.38	1.02	1130	1–5	2.30	1.01
Global Health	846	1–5	2.84	0.93	1132	1–5	2.87	0.91
Sleep Disturbances	842	1–5	2.63	1.05	1130	1–5	2.54	1.06
Burnout *	837	1–5	2.83	0.95	1129	1–5	2.74	0.97
Stress **	841	1–5	2.72	0.94	1128	1–5	2.58	0.91
Depressive Symptoms *	838	1–5	2.48	0.95	1128	1–5	2.36	0.94

* *p* ≤ 0.050; ** *p* ≤ 0.001.

**Table 6:** Psychosocial factors among study participants in 2015 and 2017—COPSOQ II scales for which the higher value are favorable.

COPSOQ II	2015				2017			
	*n*	Min–Max	Mean	SD	*n*	Min–Max	Mean	SD
Possibilities for Development	836	1–5	3.51	0.84	1126	1–5	3.55	0.82
Predictability *	846	1–5	3.05	0.95	1127	1–5	3.15	0.91
Role Clarity	843	1–5	4.05	0.76	1126	1–5	4.10	0.71
Recognition/Rewards **	841	1–5	3.68	0.92	1124	1–5	3.82	0.86
Support from colleagues	843	1–5	3.50	0.80	1127	1–5	3.53	0.79
Support from Superiors **	834	1–5	3.21	0.96	1128	1–5	3.36	0.93
Sense of Community at Work	843	1–5	4.02	0.82	1128	1–5	4.08	0.82
Quality of Leadership *	824	1–5	3.52	0.98	1109	1–5	3.66	0.92
Vertical Trust *	801	1–5	3.76	0.74	1109	1–5	3.86	0.73
Organizational Justice *	812	1–5	3.39	0.85	1109	1–5	3.48	0.85
Auto–Efficacy	840	1–5	3.96	0.67	1114	1–5	3.99	0.66
Meaning of Work *	818	1–5	3.88	0.76	1128	1–5	3.97	0.67
Workplace Commitment	845	1–5	3.23	0.89	1130	1–5	3.28	0.86
Work Satisfaction **	802	1–5	3.18	0.75	1122	1–5	3.31	0.71

* *p* ≤ 0.050; ** *p* ≤ 0.001.

**Table 7:** Distribution of the Work Ability Index (WAI) categories among study participants in 2015 and 2017.

WAI Categories		2015		2017	
		*n*	%	*n*	%
Unsatisfactory	Poor	14	1.6	19	1.6
	Moderate	140	16.2	240	20.8
Satisfactory	Good	417	48.2	554	48.0
	Excellent	294	34.0	340	29.5

**Table 8:** Logistic regression model for WAI (1 = excellent, 0 = unsatisfactory) ^1^.

Predictor	B (Coefficients)	SE (Standard Error)	Wald	df	*p*	Odds Ratio	95% C.I. Odds Ratio
Constant	−1.309	1.047	1.565	1	0.211	0.270	
Date (2017) ^2^	−0.979	0.286	11.728	1	0.001	0.376	(0.214, 0.658)
Age (≥50 years) ^3^	−0.717	0.276	6.755	1	0.009	0.488	(0.285, 0.838)
Lower-Back Symptoms (Last 12 Months) (Yes)	−1.174	0.267	19.359	1	<0.001	0.309	(0.183, 0.522)
Burnout	−1.011	0.169	35.618	1	<0.001	0.364	(0.261, 0.507)
Global Health Perception ^4^			115.422	2	<0.001		
Global Health Perception (Unfavorable)	−2.616	0.364	51.498	1	<0.001	0.073	(0.036, 0.149)
Global Health Perception (Favorable)	2.012	0.330	37.262	1	<0.001	7.481	(3.921, 14.276)
Training (Last 2 years) (Yes)	0.585	0.271	4.664	1	0.031	1.795	(1.056, 3.052)
Manual Efforts ^5^			12.921	2	0.002		
Manual Efforts (Sometimes)	0.159	0.438	0.132	1	0.717	1.172	(0.497, 2.766)
Manual Efforts (Seldom/Never)	1.117	0.372	9.002	1	0.003	3.056	(1.473, 6.339)
Sense of Community at Work	0.645	0.175	13.593	1	<0.001	1.905	(1.352, 2.684)
Meaning of Work	0.655	0.202	10.487	1	0.001	1.925	(1.295, 2.862)

^1^ Overall model evaluation (Likelihood ratio test), χ^2^ (11) = 527.507, *p* < 0.001; goodness-of-fit test (Hosmer & Lemeshow), χ^2^ (8) = 5.357, *p* = 0.719; Cox & Snell R^2^ = 0.535; Nagelkerke R^2^ = 0.731; % correct classification = 88.5% (sensitivity = 91.9%; specificity = 82.8%), AUC = 0.950 with 95% C.I. = (0.934, 0.966). ^2^ The reference category of Date is “2015”. ^3^ The reference category of Age is “<50 years”. ^4^ The reference category of Global Health Perception is “Intermediate”. ^5^ The reference category of Manual Efforts is “Frequently/Very frequently”.
